# Lower Mutation Frequency of BCP/Precore Regions in e Antigen-Negative Chronic HBV-Infected Children instead of Adults Patients

**DOI:** 10.1371/journal.pone.0120733

**Published:** 2015-03-30

**Authors:** Yong Huang, Haijun Deng, Xuefeng Shan, Xuyang Gong, Xiaosong Li, Zen Tu, Quanxin Long, Ailong Huang

**Affiliations:** 1 The Second Affiliated Hospital and the Key Laboratory of Molecular Biology of Infectious Diseases of the Chinese Ministry of Education, Chongqing Medical University, Yuzhong, Chongqing, China; 2 Department of Clinical Laboratory, Second Affiliated Hospital, Chongqing Medical University, Yuzhong, Chongqing, China; 3 The Department of Pharmacy, the First Affiliated Hospital, Chongqing Medical University, Yuzhong, Chongqing, China; 4 Collaborative Innovation Center for diagnosis and treatment of infectious diseases, Hangzhou, China; CRCL-INSERM, FRANCE

## Abstract

To describe the Hepatitis B e antigen(HBeAg) seroconversion related mutation profiles of the basal core promoter(BCP)/precore regions in e antigen seroconverted child patients, a cohort of 245 child patients with CHB and a control patients group of 92 adult patients with CHB were recruited. The mutation frequencies of six nucleotides or nucleotide combinations including nucleotide (nt)1896, nt1762/1764, nt1752, nt1846, nt1899 and nt1753 showed significant differences between HBeAg positive and HBeAg-negative child patients groups. The frequencies of these HBeAg seroconversion-related mutations were significantly lower in HBeAg-negative children with CHB than in HBeAg-negative adults with CHB, especially for the mutation G1896A (41.1% *vs* 91.7%, *P*<0.001), and the average number of BCP/precore region mutations in samples from HBeAg-negative child patients was also obviously lower than in HBeAg-negative adult patients(3.62±3.03 *vs* 4.89±2.09, *P*<0.001), suggesting less impact of mutations in the BCP/precore region on HBeAg seroconversion in child patients than adult patients.

## Introduction

Chronic hepatitis B virus(HBV) infection(CHB) leads to a continuum of clinical outcomes, ranging from an asymptomatic carrier state to chronic active hepatitis, cirrhosis, and hepatocellular carcinoma[1]. Although the prevalence of HBV infection in children and adolescents decreased dramatically due to the universal HBV vaccination plan recommended by the World Health Organization(WHO) beginning in 1991[2, 3], more than 240 million people remain chronic liver infected, representing a serious global health problem[4].

The natural history of CHB can be divided into four phases: the immune tolerant phase, the immune active phase, the inactive phase and reactivation[5–7]. hepatitis B e antigen (HBeAg) seroconversion, defined as the loss of HBeAg followed by gain of anti-HBe antibodies, is an important hallmark of HBV natural infection history[8],suggesting the transition from an active phase to an inactive phase. Previous studies have demonstrated that HBeAg seroconversion in HBeAg-positive CHB patients is accompanied by improved clinical outcomes, and current clinical management guidelines have adopted HBeAg seroconversion as an appropriate treatment end point for CHB patients[9–11].

HBeAg seroconversion can be defined as a balance point in the battle between HBV and the host immune response. G1896A and the A1762T and G1764A double mutation are the most frequent isolated mutations in the precore and basal core promoter (BCP) regions of the HBV genome isolated from HBeAg-negative patients. The mutation G1896A creates a premature stop codon (28^th^ codon) that prevents the production of HBeAg [12, 13], and the A1762T and G1764A double mutation in the BCP region can reduce the synthesis of HBeAg and enhance viral replication [14]. These mutation in BCP/precore regions were significantly associated with aggressive hepatitis and advanced liver disease[15].

Most HBeAg seroconversion factors were largely studied in adult patients[16, 17], the relationship between HBeAg seroconversion and BCP/precore region mutations in infants and children with CHB was less addressed[18, 19]. One previous study revealed that spontaneous HBeAg seroconversion usually occurred after puberty, with approximately 90% of children <15 years of age still HbeAg positive[20], suggesting possible difference between natural seroconversion rates in child and adult patients. From a population age perspective, sequences from HBeAg-negative child patients is much closer to seroconversion time point than that from HBeAg-negative adult patients, and sequences may more truly reflect HBeAg seroconversion-related feature. In this study, we performed a cross-sectional study to analyze the mutation profiles of the BCP/precore regions in a large number of well-defined children patients and adult control patients.

## Materials and Methods

### Subjects

A total of 337 serum samples were obtained from the Children’s Hospital of Chongqing Medical University, the first affiliated hospital of Chongqing Medical University, the second affiliated hospital of Chongqing Medical University, Guangzhou Women and Children’s Medical Center and Guangdong Women and Children’s Hospital between June 2011 and September 2013 and stored at -70°C until further testing. In detail, all sera from HBeAg negative child CHB patients admitted between June 2011 and September 2013(110 samples) were collected, 90 of these samples were successfully amplified. Another 155 HBeAg positive child patients and 92 adult patients were recruit as controls.

All of the patients, including245 children and 92 adults with chronic HBV infection, were positive for hepatitis B surface antigen (HBsAg) but negative for anti-HBs, anti-HCV(Hepatitis C virus), anti-HDV(Hepatitis D virus)and anti-HIV(Human Immunodeficiency Virus) antibodies. The Chongqing Medical University ethics committee approved the study, and written informed consent for participation in this study was obtained from all of the adult patients or the caretakers on behalf of the children enrolled in this study.

### Serological Investigations and HBV DNA Quantification

HBsAg, anti-HBs, HBeAg, anti-HBe and anti-HBc levels were determined using enzyme-linked immunosorbent assay(ELISA, KeHua) or radioimmunoassay(RIA) kits(Roche). The liver function profile was determined using an auto-analyzer (Hitachi 7170, Tokyo, Japan). HBV viral load was assessed by real-time polymerase chain reaction (PCR) using a fluorescence quantitative(FQ)-PCR kit for HBV (DaAn Gene Co., China) according to the manufacturer’s instructions.

### Amplification and Sequencing of BCP/precore regions of the HBV genome

Serum HBV DNAextraction, BCP/precore region(nt1653-1959) amplifications and sequencing followed previous descriptions[21]. Accession numbers of BCP/precore regions sequences from child patients with CHB were from KP309449 to KP309693, and that of adult patients with CHB were from KP309694 to KP309785.

### Mutations and Genotype Analysis

Mutations of the BCP/precore regions were determined by aligning the sequences of the clinical samples with their corresponding consensus sequences for genotype B or genotype C. Consensus sequences for the different genotypes of HBV were calculated using the Basic Local Alignment Search Tool(BLAST) software with 2600 complete HBV genome sequence from GenBank. The complete S gene (nt65 to 870) of 337 samples was amplified by nested PCR as previously described[22] and then analyzed using the National Center for Biotechnology Information (NCBI) genotyping tool.

### Statistical Analysis

The chi-squared test and Fisher’s exact test were used to determine the differences between groups for categorical variables such as frequency of mutation. The Wilcoxon-Mann-Whitney test was used for continuous variables, such as alanine aminotransferase (ALT) level, DNA load, and the number of nucleotide mutations. Logistic linear regression analysis was used to assess the correlation between the number of nucleotide mutations and the HBeAg-negative subjects ratio. A *P* value of< 0.05 was considered statistically significant. All of the tests were analyzed using the Statistical Package for Social Science(SPSS) version 17.0 software.

## Results

### Clinical information of samples

A total of 245 children with CHB and 92 adults with CHB were enrolled in this cross-sectional study. The percentage of HBeAg-negative samples in the children and adult patients was 36.7% and 52.1%, respectively. The average age of the HBeAg seroconverted patients was older than the average age of the HBeAg-positive patients in both groups (7.93±3.82 *vs* 5.66±3.94 in children, 42.48±9.05 *vs* 35.05±8.10 in adults, *P* <0.001 for both), suggesting that HBeAg seroconversion is positively correlated with age(Table 1). The serum HBV DNA titers of the HBeAg-negative patients were obviously lower than that of antigen-positive patients. There were no significant differences in virus genotype, gender or ALT levels between HBeAg-negative and-positive child or adult patients.

**Table 1:** The clinical information of chronic hepatitis B patients.

	HBeAg(+) Children N = 155	HBeAg(-) Children N = 90	*P* value	HBeAg(+)adultN = 44	HBeAg(-) adultN = 48	*P* value
Sex: Male/Female	92/63	57/33	0.632	31/13	35/13	0.976
Age (years)	5.66±3.94	7.93±3.82	<0.001	35.05±8.10	42.48±9.05	<0.001
ALT (IU/L)	73.27±148.88	60.17±111.63	0.436	82.9±86.94	81.3±98.2	0.834
HBV DNA(log10 copies/ml)	8.02±1.16	4.25±1.88	<0.001	8.04±0.95	5.99±1.49	<0.001
Genotype: B/C	116/39	75/15	0.166	27/3	25/6	0.504

### Mutation sites correlated with HBeAg seroconversion

**Fig 1:**
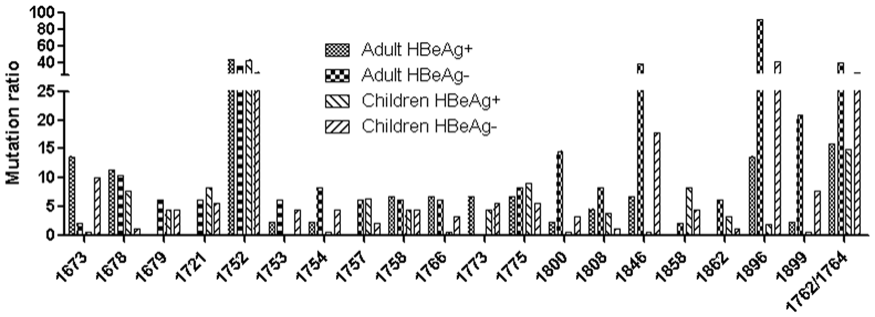
Mutation sites with >5% ratio in BCP/precore region.

As shown in Fig. 1, there were 20 nucleotides with mutation rates higher than 5%. Among them, six nucleotide or nucleotide combinations, including nt1752, nt1753, nt1762/1764, nt1846, nt1896, and nt1899,showed a significant difference between the HBeAg-positive and-negative child patient groups (Table 2). Moreover, only four sites or site combinations(nt1846, nt1896, nt1899 and nt1762/1764) showed a significant difference in the mutation ratio between the HBeAg-positive and-negative adult patient groups. Notably, the frequency of the G1896A mutation in the HBeAg-negative child patients was significantly lower than in the HBeAg-negative adult patients (41.1% *vs* 91.7%, *P* < 0.001), and similar results were also observed for other BCP/precore mutations such as A1846T (17.8% *vs* 37.5%, *P* = 0.010) and the G1899A substitution (7.8% *vs* 20.8%, *P* = 0.026). The mutation frequencies for nt1762/1764, nt1752 and nt1753 in HBeAg-negative child patients were also lower than in e negative-adult patients, although the difference lacked statistical significance.

**Table 2:** Mutation sites that correlated with the HBeAg status in children patients and adult patients.

Mutation sites	Children patients, n = 245		*P*_1_ *value*	Adult patients,n = 92		*P*_2_ *value*	*P*_3_ *value*
	HBeAg +, n = 155	HBeAg-, n = 90		HBeAg +, n = 44	HBeAg-, n = 48		
1752	42.6%, 66/155	27.8%, 25/90	0.029	43.2%, 19/44	35.4%, 17/48	0.446	0.353
1753	0.0%, 0/155	4.4%, 4/90	0.034	2.3%, 1/44	6.3%, 3/48	0.342	0.958
1846	0.6%, 1/155	17.8%, 16/90	<0.001	6.8%, 3/44	37.5%, 18/48	<0.001	0.010
1896	1.9%, 3/155	41.1%, 37/90	<0.001	8.2%, 6/44	91.7%, 44/48	<0.001	<0.001
1899	0.6%, 1/155	7.8%, 7/90	0.008	2.3%, 1/44	20.8%, 10/48	0.006	0.026
1762/1764	14.8%,23/155	26.7%, 24/90	0.023	15.9%, 7/44	39.5%, 19/48	0.012	0.119

*P*_1_ represented the statistics difference of certain site mutation ratio between the HBeAg positive children patients and HBeAg negative children patients.*P*_*2*_ represented the statistics difference of certain site mutation ratio betweenHBeAg positive adult patient andHBeAg negative adult patients. *P*_*3*_represented the statistics difference of certain site mutation ratio betweenHBeAg negative children patients and HBeAg negative adult patients.

Several HBeAg seroconversion-related mutation sites were also significantly correlated with the HBV genotype (Table 3). The frequency of the A1752G mutation was higher in genotype B than genotype C HBV (46.1% *vs* 5.6% in children, 53.0% *vs* 3.8% in adults, *P*<0.001 for both). In comparison, the frequencies of the A1762T/G1764A double mutation and T1753V mutation increased in genotype C infected children patients (*P*< 0.05).

**Table 3:** The mutation profiles/ frequencies of HBeAg seroconversion related mutation nucleotides site in different HBV genotypes.

Mutation sites	Genotype B	Genotype C	Children patients		*P*_*1*_value	Adult patients		*P*_*2*_value
			B (n = 191)	C (n = 54)		B (n = 66)	C (n = 26)	
1752	A1752G	A1752G	46.1%	5.6%	0.000	53.0%	3.8%	0.000
1753	T1753V	T1753V	0.5%	5.6%	0.035	1.5%	11.5%	0.120
1846	A1846T	A1846T	7.9%	3.7%	0.449	23.4%	23.1%	0.971
1896	G1896A	G1896A	18.3%	9.3%	0.112	57.6%	46.2%	0.322
1899	G1899A	G1899A	2.6%	5.6%	0.523	10.6%	15.4%	0.780
1762/1764	A1762T/G1764A	A1762T/G1764A	15.7%	31.5%	0.009	24.2%	43.5%	0.081

*P*_1_ represented the statistics difference of certain site mutation ratio between the genotype Band genotype C in children patients.*P*_*2*_represented the statistics difference of certain site mutation ratio between the genotype Band genotype C in adult patients.

### Combined mutation profiles of BCP/precore regions in HBeAg seroconverted child and adult patients

There were 127 combined mutation types in the child and adult patients with CHB; among them, 10 mutation types showed a differential distribution between HBeAg-negative and HBeAg-positive samples (Table 4). The ratio of three combined mutations(G1896A/A1762T/G1764A, G1896A/A1846T and G1896A/A1752G)was obviously higher in HBeAg-negative patients than in the HBeAg-positive patient groups, suggesting that these combined mutation types could distinguish the HBeAg seroconverted patients with CHB. Notably, the higher mutation ratio in the adult patients than in the child patients for these combined mutations (G1896A/A1846T: 35.4%*vs*12.2%, *P*<0.001; G1896A/G1899A: 18.8%*vs*4.6%, *P* = 0.015; G1896A/A1762T/G1764A: 35.4%*vs*12.2%, *P*<0.001) implied that combined mutations in the BCP/precore regions were less correlated with HBeAg seroconversion in child patients than adult patients.

**Table 4:** Combined mutations profilesof BCP/precorein the HBeAgseroconversion children and adult patients.

combined mutation types	children patients		*P*_1_ *value*	adult patients		*P*_2_ *value*	*P*_3_ *value*
	HBeAg+, n = 155	HBeAg-, n = 90		HBeAg+, n = 44	HBeAg-, n = 48		
1896/1846	0	12.2	0.000	4.5	35.4	0.000	0.001
1896/1899	0	4.6	0.017	0	18.8	0.008	0.015
1896/1762/1764	0.6	12.2	0.000	2.3	35.4	0.000	0.001
1896/1752	1.3	12.2	0.001	4.5	35.4	0.000	0.001
1899/1762/1764	0	5.9	0.006	0	12.5	0.045	0.269
1899/1896/1846	0	3.4	0.099	0	12.5	0.045	0.086
1899/1896/1762/1764	0	3.4	0.049	0	10.4	0.082	0.189
1752/1896/1762/1764	0.6	4.4	0.062	0	18.8	0.008	0.015
1762/1764/1846/1899/1896	0	2.3	0.134	0	6.3	0.272	0.467
1896/1846/1762/1764	0	2.3	0.134	2.3	14.6	0.085	0.015

Mutation ratio was the ratio of the number of specific linkage mutation type to the total sample number. *P*_*1*_ value, *P*_*1*_ represented the statistics difference of certain linkage mutation ratio between the HBeAg positive children patients and HBeAg negative children patients. *P*_*2*_ represented the statistics difference of certain linkage mutation ratio between HBeAg positive adult patient and HBeAg negative adult patients. *P*_*3*_ represented the statistics difference of certain linkage mutation ratio between HBeAg negative children patients and HBeAg negative adult patients.

### Correlation analysis of BCP/precore region mutation numbers and HBeAg serological status

**Fig 2:**
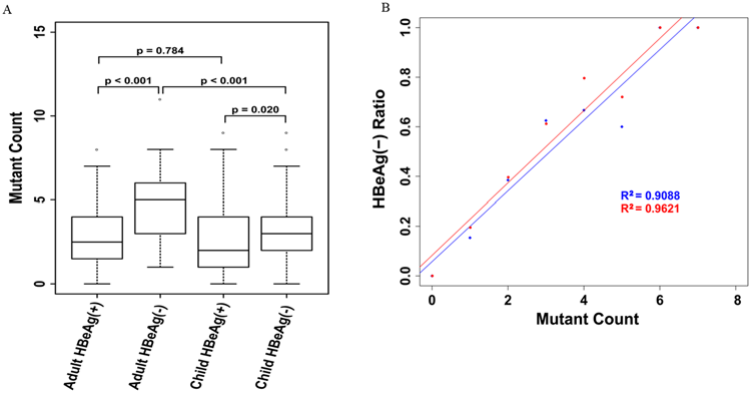
The relationship between number of mutation in BCP/precore region and e antigen seroconversion status. A. Average number of mutations nucleotides in BCP/precore region in children and adultpatients with CHB, Average number of mutations in BCP/precore regions of HBeAg negative patients were obviously higher than in HBeAg positive patients either in adult and children patients group; Average number of mutations in BCP/precore regions of adult HBeAg negative patients was also statistical higher than that of children HBeAg negative patients. B. Correlations between the number of mutant sites in BCP/precore and HBeAg seroconversion.red line representedthe correlation between BCP/precore mutation count and HBeAg negative ratio in children patients, *P*<0.001; blue line represented the correlation between precore/core mutation count and HBeAg negative ratio in adult patients, *P*<0.001.

In the present study, the average number of mutations in the BCP/precore regions of the HBeAg-negative subjects was significantly higher than in the HBeAg-positive subjects in both the child and adult patient groups (3.62±3.03 *vs* 2.88±1.94, *P* = 0.020 in child patients; 4.89±2.09 *vs* 2.91±1.88, *P*< 0.001 in adult patients) (Fig. 2a), directly proving that the number of mutations was positively related to HBeAg seroconversion. In addition, the average number of mutations in this region in HBeAg-negative child patients was also obviously lower than in HBeAg-negative adult patients(3.62± 3.03 *vs* 4.89± 2.09, *P*<0.001). Samples were also grouped according to the number of mutations in the BCP/precore region, and the HBeAg seroconversion ratio was calculated for each group. A significant positive correlation between the number of mutations and HBeAg seroconversion was observed in our samples (R^2^ = 0.9088 in children, R^2^ = 0.9621 in adults, *P*< 0.05 for both) (Fig. 2b), and the relevance level in adult patients was also higher than in children, confirming that the aforementioned BCP/precore mutations are less important for children who HBeAg seroconvert.

### The association of HBV BCP/precore mutations with clinical presentation in chronic HBV patients

**Fig 3:**
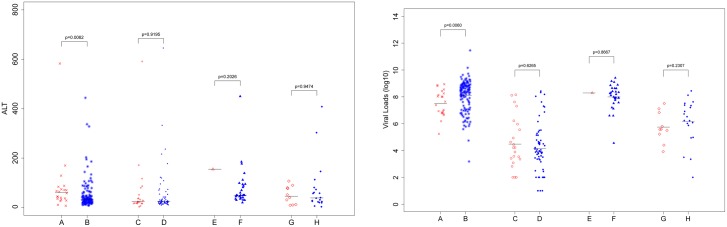
The association analysis between A1762T/G1764A double mutation and viral load, ALT level in different chronic HBV patients. A. children HBeAg positive patients containing A1762T/G1764A double mutation; B. children HBeAg positive patients not containing double mutation; C. children HBeAg negative patients containing double mutation; D. children HBeAg negative patients not containing double mutation; E. adult HBeAg positive patients containing double mutation; F. adult HBeAg positive patients not containing double mutation; G. adult HBeAg negative patients containing double mutation; H. adult HBeAg negative patients not containing double mutation.

The association between 20 hotspot mutation sites and the patients’ clinical presentations, including viral DNA titers, ALT levels and ages, was analyzed. Most mutations had no correlation with the clinical phenotypes, except the A1762T/G1764A double mutation, which was significantly correlated with a low viral DNA load and high ALT levels in HBeAg-positive child patients (viral load log_10_8.10±1.17 *vs* log_10_7.51±0.97, *P* = 0.006 and ALT 59.3±114.7 *vs* 129.7±242.4, *P* = 0.006 for wildtype group *vs* double mutation group, respectively), but this correlation was not present in the HBeAg-negative child patient group (Fig. 3). In HBeAg-positive adult CHB patients, the A1762T/G1764A double mutation was significantly correlated with a low viral load (log_10_7.39±1.36 *vs* log_10_6.15±1.59,wild type group *vs* double mutation group, *P* = 0.0077) but not with ALT level (89.6±101.8 *vs* 317.5±229.8, wild type group *vs* double mutation group, respectively, *P* = 0.0681), which differs from previous findings[23]. This difference is most likely due to the small sample size of our HBeAg-positive adult patients(Fig. 3).

## Discussion

Two mutations in BCP/precore region were frequently associated with HBeAg seroconversion. One variation is a G-to-A mutation at nt1896, which creates a premature stop codon and then abolishes the synthesis of HBeAg[24]. The other is a two-nucleotide substitution, T1762A and G1764A[25], and transfection studies have shown that the T1762A and G1764A double mutation decreases the level of pre-C mRNA by 50% to 70%, leading to reduced HBeAg synthesis[26, 27]. In these six HBeAg seroconversion statistics-related sites, G1896A and the A1762T/G1764A double mutation had obviously higher mutation ratios in HBeAg-negative patients, which is in accordance with previous studies[12, 28]. In our study, the nt A1846T mutation ratio in HBeAg-negative child and adult patients was also significantly higher than in HBeAg-positive child and adult patients(17.8% *vs* 0.6%,*P*<0.01, 37.5% *vs* 6.8%, *P*<0.01 for children and adults, respectively), providing a clue that A1846T is closely associated with HBeAg seroconversion. The wild-type HBV sequence at position 1846 is T in most common genotypes, but it is A in the genotypes B and C that are prevalent in China(29). A1846T mutation is silent at the amino acid level, and previous studies have observed that this mutation is associated with the progression of age-dependent disease (liver cirrhosis and hepatocellular carcinoma) as well as severe liver disease (fulminant hepatitis and acute-on-chronic liver failure, ACLF) [29, 30]; however, the relationship between this mutation and HBeAg seroconversionneeds further experimental validation. Although nt1753 mutation has been reported as a rarity[31], significantly higher mutation ratio have also been observed in HBeAg-negative child patients. Combined mutations at nt1753, nt1762, nt1764, and nt1766 contribute to lower levels of HBeAg expression than double mutations at nt1762 and nt1764[32], which suggests that nt1753 mutation is most likely related to lower HBeAg expression levels. Although the G1899A mutation only changes the glycine at codon 29 into aspartic acid and is always accompanied by the G1896A mutation, a previous study revealed that approximately 50% of the HBeAg negative variants contained this combined mutation[33]. Interestingly, the frequency of the A1752G mutation was significantly higher in HBeAg-positive child patients(42.6% *vs* 27.8%, *P*<0.05)but was negatively correlated with HBeAg seroconversion. This statistically significant difference was not observed in adult patients. And the older patients possessed higher HBeAg seroconversion ratio, consistent with previous reports[20].

Due to limitations in sample size for cross-sectional studies, previous studies have rarely attempted to analyze the mutation frequency discrepancy between HBeAg negative child patients and HBeAg negative adult patients[34]. It is worth noting that the mutation ratio of G1896A is obviously lower in HBeAg-negative child(41.1%) than in HBeAg-negative adult patients(91.7%)(*P*<0.001). Similar features were also observed for other sites located in the BCP/precore regions(G1899A and A1846T, *P*<0.05)(Table 2). These difference suggest the possibility that mutations in the BCP/precore region have less impact on HBeAg seroconversion in child patients than in adult patients. In addition to mutations in the BCP/precore region, deletions in core regions may also have an impact on HBeAg expression. Previous studies have demonstrated that core gene mutants appear during the HBeAg clearance phase[35], and a long-term, large-scale cohort study carried out in children also showed the core mutations that most strongly signified HBeAg seroconversion within 1 year, and the core deletion mutations disappeared after HBeAg seroconversion[36]. Deletions in core regions instead of the G1896A and A1762T/G1764A double mutation were observed in early seroconversion processes in a longitudinal study of infants (our own unpublished data), which also supports the idea that HBeAg seroconversion does not solely rely on BCP/precore mutations. Mutation or deletions in core regions were apt to occur due to host immune stress because core particles are the major carriers of T/B cell epitopes[37], but the fitness cost of core protein silencing was higher due toits essential roles in virus nucleocapsid assembly[38]. In combination with the lower mutation frequency of the BCP/precore regions in HBeAg-negative child patients, we hypothesize that deletions in core regions that were screened by the host immune response were the major reason for the initial phase of HBeAg seroconversions, but due to its high fitness cost, BCP/precore mutations were substituted for core region mutations in the latter phase of HBeAg seroconversion. Sequential nucleotide acid variations in the core regions and BCP/precore regions may explain HBeAg seroconversion.

The relationship between the A1762T/G1764A double mutation and the level of viral DNA replication level is unclear, and studies that employed the transfection of human hepatoma cell lines with clinical HBV viral samples may not truly reflect the true *in vivo* status[26, 39–43]. In our cross-sectional study, BCP double mutations were associated with a lower viral load and an elevated ALT level in HBeAg-positive child patients. There were high levels of viral replication during the immune tolerance phase, while the HBV DNA levels fluctuated and progressively decreased in the immunoreactive phase. The HBeAg remained positive until cleared by the immune system at the end of the second phase. HBeAg has the potential to preferentially deplete inflammatory HBeAg- and HBcAg-specific Th1 cells that are necessary for viral clearance, therefore promoting HBV persistence[44]. An A1762T/G1764A double mutation in the BCP/precore region would reduce the levels of HBeAg by inhibiting precore mRNA expression[45], leading to the transition from the immune tolerance to the immune reactive phase, followed by lower DNA viral loads and elevated ALT levels. The reasons for the lower viral loads with A1762T/G1764A double mutations in HBeAg-positive patients were more likely related to the decreased HBeAg synthesis and alterations in the complex delicate balance between the virus and the host immune system, but the alterations cannot simply be attributed to reduced viral replication mediated by the double mutation.

## Conclusions

The mutation ratios of nt1752, nt1753, nt1762/1764, nt1846, nt1896and nt1899were significantly different between the HBeAg-positive and-negative child patients groups, and the frequencies of these mutations in HBeAg-negative child patients were significant lower than in HBeAg-negative adult patients, implying that the role of BCP/precore mutations is less important in the early phases of HBeAg seroconversion. The number of mutations in the BCP/precore regions was also positively correlated with the HBeAg negative ratio. The correlation analysis between the mutations and the clinical features revealed only that thent1762/1764 double mutation was associated with lower viral loads and higher ALT levels in HBeAg-positive children with CHB.
